# Survival prediction for Philadelphia chromosome-like acute lymphoblastic leukemia by machine learning analysis: a multicenter cohort study

**DOI:** 10.3389/fcell.2025.1650810

**Published:** 2025-09-18

**Authors:** Xiao-Dan Song, Dan-Na Lin, Lv-Hong Xu, Li-Ying Liu, Chi-Kong Li, Xiao-Rong Lai, Ya-Ting Zhang, Wu-Qing Wan, Xiao-Li Zhang, Xiang Lan, Xing-Jiang Long, Bei-Yan Wu, Qi-Wen Chen, Li-Hua Yang, Yun-Yan He

**Affiliations:** ^1^ Graduate School, Guangxi Medical University, Nanning, China; ^2^ Department of Pediatric Hematology, Zhujiang Hospital, Southern Medical University, Guangzhou, China; ^3^ Department of Pediatrics, Sun Yat-sen Memorial Hospital, Sun Yat-sen University, Guangzhou, China; ^4^ Department of Pediatrics, Prince of Wales Hospital, The Chinese University of Hong Kong, Hong Kong SAR, China; ^5^ Division of Hematology and Tumor, Children’s Medical Center, the Second Xiangya Hospital, Central South University, Changsha, China; ^6^ Department of Pediatrics, The First Affiliated Hospital, Sun Yat-sen University, Guangzhou, China; ^7^ Department of Pediatrics, Affiliated Hospital of Guangdong Medical University, Zhanjiang, China; ^8^ Department of Pediatrics, Liuzhou people’s hospital, Liuzhou, China; ^9^ Department of Pediatrics, The First Affiliated Hospital of Shantou University Medical College, Shantou, China; ^10^ Department of Pediatrics, The First Affiliated Hospital of Nanchang University, Nanchang, China; ^11^ Department of Pediatrics, The First Affiliated Hospital of Guangxi Medical University, Nanning, China

**Keywords:** Philadelphia chromosome-like acute lymphoblastic leukemia, machine learning, random forest, minimal residual disease, survival prediction

## Abstract

**Background:**

This study aimed to develop an efficient survival model for predicting event-free survival (EFS) in patients with Philadelphia chromosome (Ph)-like acute lymphoblastic leukemia (ALL).

**Methods:**

Data related to Ph-like ALL were collected from the South China Children’s Leukemia Group (SCCLG) multicenter study conducted from October 2016 to July 2021. A model for predicting the survival of patients with Ph-like ALL was built using Cox proportional hazards regression, random forest, extreme gradient boosting, and gradient boosting machine techniques. By integrating indicators including the concordance index (C-index), 1-, 3-, and 5-year area-under-the-receiver operating characteristics curve (AUROC), Brier score, and decision curve analysis, the predictive capabilities of each model were compared.

**Results:**

The random forest algorithm demonstrated the most robust predictive performance. In the test set, the C-index of the random forest model was 0.797 (95% CI: 0.736–0.821; P < 0.001). The AUROCs for 1, 3, and 5 years were 0.787 (95% CI: 0.62–0.953; P < 0.001), 0.797 (95% CI: 0.589–1; P < 0.001), and 0.861 (95% CI: 0.606–1; P < 0.001), respectively. The Brier scores for 1, 3, and 5 years were 0.102 (95% CI: 0.032–0.173; P < 0.001), 0.126 (95% CI: 0.063–0.19; P < 0.001), and 0.121 (95% CI: 0.051–0.19; P < 0.001), respectively.

**Conclusion:**

The random forest model effectively predicted the survival outcomes of patients with Ph-like ALL, which can aid clinicians to conduct personalized prognosis assessments in advance. Based on a web-based calculator, using random forest prediction models to calculate the prognosis of Ph-like ALL (https://songxiaodan03.shinyapps.io/RFpredictionmodelforPHlikeALL/) could facilitate healthcare professionals in carrying out clinical evaluation.

## 1 Background

Philadelphia chromosome (Ph)-like acute lymphoblastic leukemia (ALL), a subtype of acute lymphoblastic leukemia, exhibits gene expression profiles and activated kinase signaling pathways similar to those of Ph + ALL yet notably lacks the BCR-ABL1 fusion gene (Harvey and Tasian, 2020; Yadav et al., 2021; Roberts, 2017). Although research indicates that ABL fusion and activation of the JAK-STAT signaling pathway are commonly present in this subtype (Roberts et al., 2017a; Roberts et al., 2012), some cases of Ph-like ALL do not have typical identifiable genetic abnormalities; this, to some extent, limits diagnostic accuracy based on traditional cell morphology and immunophenotypes (Tran and Tasian, 2022; Schwab and Harrison, 2018; Anagnostou et al., 2020; Tasian et al., 2017). Compared with cases involving typical fusion genes, this situation renders the diagnosis and prognosis of Ph-like ALL more challenging. There are significant international differences in the incidence rate of Ph-like ALL. In pediatrics, the incidence rate ranges from 5% to 30% (Chiaretti et al., 2019; Hu et al., 2023; Roberts et al., 2018), and the prognosis is relatively poor (Al Ustwani et al., 2016; Frisch and Ofran, 2019; Roberts et al., 2017b; Shiraz et al., 2020). The incidence rate of the disease also increases with age (Herold et al., 2014), accounting for 15%, 21%, and 27% of children, adolescents, and young adults, respectively, with B-cell acute lymphoblastic leukemia (B-ALL) (Roberts et al., 2014a).

Current research on Ph-like ALL involves numerous complex scenarios that are influenced by multiple genetic variations and treatment outcomes under different treatment regimens (Roberts et al., 2017b; Iacobucci and Roberts, 2021; Abou Dalle et al., 2019). For instance, various intensified chemotherapy regimens or combined tyrosine kinase inhibitors are employed to enhance the survival outcomes and optimize management strategies for patients with Ph-like ALL, primarily due to the poor prognosis of the disease. Arber, Roberts, and other researchers posit that intensified therapies (including transplantation) are pivotal in treating Ph-like ALL. Although some patients demonstrate higher levels of minimal residual disease (MRD) at the end of induction therapy, their survival rates remain comparable to those of non-BCR/ABL1-like ALL patients (Arber et al., 2016; Roberts et al., 2014b). Stock et al. (2019) and Chiaretti et al. (2021) found that the genetic molecular features of Ph-like ALL are associated with lower survival rates and pose a risk factor for event-free survival (EFS) in these patients. However, other large-scale clinical trials have reached different conclusions. After analyzing the clinical trial data of the Australian and New Zealand Children’s Hematology/Oncology Group (ANZCHOG) ALL8, Heatley et al. (2017) discovered that even with risk-stratified treatment based on MRD evaluation, the recurrence of Ph-like ALL in children remained high, which in turn affected the EFS time. Therefore, research on the prognosis of Ph-like ALL is still in progress and aims to clarify the true factors affecting it. Effective prognostic assessment can accurately determine which patients can benefit from aggressive treatment options such as bone marrow transplantation and which patients may be more suitable for standard treatment (Yadav et al., 2021), thereby providing them with more precise and personalized treatment options.

Machine learning, a form of artificial intelligence, comprises various algorithms. These algorithms can continuously improve performance through iteration and make predictions analogous to human decisions (Handelman et al., 2018). In recent years, the application of machine learning in the medical field has become increasingly prevalent, particularly in processing large clinical datasets to address complex medical problems, with significant advantages in high-dimensional data processing, nonlinear model construction, and predictive algorithm development. Existing research has demonstrated that it is feasible to use machine learning for deep learning analyses of lesion imaging patterns and immunohistochemical presentations, combined with clinical indicators or omics data for early disease diagnosis and computational analyses (Handelman et al., 2018; Choi et al., 2020; Greener et al., 2022).

Based on the above circumstances, integrating machine learning modeling to predict the prognosis of Ph-like ALL is feasible. This study intends to utilize random forest (RF), gradient boosting machine (GBM), extreme gradient boosting (XGBoost), and traditional Cox hazard regression algorithms to analyze the genetic and clinical features of Ph-like ALL and construct a model for predicting the survival rate of the disease. The optimal prediction model was determined by comparing the performance of each model.

## 2 Methods

### 2.1 Study subjects and data collection

#### 2.1.1 Study subjects

A retrospective multicenter study was conducted from October 2016 to July 2021 on children newly diagnosed with ALL at 13 affiliated hospitals participating in the South China Children’s Leukemia Group (SCCLG)-ALL-2016 multicenter study. The treatment protocol of this study strictly adhered to the SCCLG-ALL-2016 guidelines for ALL (version 20191101.5). This study was reviewed and approved by the Ethics Committee of Sun Yat-sen Memorial Hospital, Sun Yat-sen University. All research was conducted in accordance with the International Code of Medical Ethics of the World Medical Association (Declaration of Helsinki). Additionally, this study was registered in the Chinese Clinical Trial Registry (Chi-CTR; https://www.chictr.org.cn/; registration number ChiCTR2000030357).

#### 2.1.2 Inclusion and exclusion criteria for the SCCLG-ALL-2016 multicenter study

The inclusion criteria (Harvey and Tasian, 2020) required participants to be 18 years old or younger (Yadav et al., 2021). Based on the 2008 World Health Organization classification criteria, in combination with various results such as bone marrow smear morphology, immune phenotype, cytogenetics, and molecular genetics, the clinical manifestations were consistent with ALL, and patients were diagnosed with B-ALL (Roberts, 2017). Patients must be pediatric patients experiencing their first episode of the disease.

Exclusion criteria (Harvey and Tasian, 2020) comprised patients with T-lineage leukemia, mature B-cell leukemia, or acute mixed leukemia (Yadav et al., 2021), patients with secondary leukemia resulting from immunodeficiency (Roberts, 2017), patients with a history of a second malignancy (Roberts et al., 2017a), patients with Down syndrome (Roberts et al., 2012), and patients who had used glucocorticoids for more than 1 week within the month prior to enrollment.

#### 2.1.3 Diagnostic and exclusion criteria for Philadelphia chromosome-like acute lymphoblastic leukemia

According to the International Consensus Classification (ICC) of ALL, gene expression profiling is the most reliable approach for diagnosing Ph-like ALL. When comprehensive gene expression profiling testing was unavailable, techniques such as fluorescence *in situ* hybridization, polymerase chain reaction (PCR), reverse transcriptase PCR, flow cytometry, transcriptome sequencing, and whole-exome sequencing were employed to identify fusion genes (e.g., CRLF2, JAK2, EPOR, ABL1, ABL2, and PDGFRB) to aid in clinical diagnosis. Specific probes were used to detect common genetic abnormalities in Ph-like ALL, including rearrangements and mutations in genes such as SH2B3, JAK1/2/3, and IL7R. The exclusion criteria for Ph-like ALL were as follows: ALL patients who did not meet the diagnostic criteria for Ph-like ALL, Ph-like ALL patients who were lost to follow-up, and Ph-like ALL patients who lacked the aforementioned basic clinical data.

#### 2.1.4 Chemotherapy regimen

Treatment for Ph-like ALL patients was implemented according to the SCCLG-ALL-2016 protocol. At the time of enrollment, prednisone was initially administered for a 7-day pretreatment period, followed by diagnosis and sensitivity assessment. Subsequently, remission induction therapy based on vincristine, dexamethasone, L-asparaginase, and daunorubicin (VDLD) was performed, along with early intensified cyclophosphamide, cytarabine, 6-mercaptopurine, and L-asparaginase (CAM + L-asparaginase) therapy. The consolidation regimen utilized high-dose methotrexate and 6-mercaptopurine, while the re-induction phase employed a delayed intensive VDLD regimen combined with CAM + L-asparaginase. Maintenance therapy included chemotherapy and regular intrathecal injections, with specific dosages and risk assessment criteria detailed in Li et al. (2021) and Radu et al. (2020).

#### 2.1.5 Indications for transplantation and tyrosine kinase inhibitor regimen

Indications for transplantation encompassed the following scenarios: failure to attain remission after induction therapy (i.e., bone marrow morphology failed to meet remission criteria on day 33); MRD level ≥10^−4^ prior to consolidation treatment (week 12); early bone marrow relapse (occurring within 6 months after treatment cessation or within 36 months of diagnosis). Regarding the TKI regimen, once a patient was diagnosed with Ph-like ALL, dasatinib or imatinib was incorporated into the standard chemotherapy regimen starting on the 15th day of induction therapy, and the treatment was continued until the maintenance phase.

#### 2.1.6 Clinical data collection

Clinical characteristics data of pediatric patients were collected utilizing electronic medical record systems from multiple hospitals. These data included gender, age, peripheral blood leukocyte count at diagnosis, peripheral blood platelet count at diagnosis, hemoglobin concentration at diagnosis, proportion of bone marrow primitive white blood cells plus immature white blood cells (blasts) at diagnosis, chromosome morphology classification, immunophenotypes (Pro-B-ALL, common B-ALL, Pre-B-ALL, mixed phenotype B-ALL, immature B-ALL), extramedullary tumor (ET), IK6 mutation or deletion, ABL fusion, kinase pathway dysregulation, prednisone response (PR) on day 8 (sensitive: peripheral blood blasts <1 × 10^9^/L, insensitive: peripheral blood blasts >1 × 10^9^/L), bone marrow response rate on day 15 (D15 BMR; M1: ratio of primitive cells to naive cells <5%, M2: 5%–25%, M3: >25%, M4: bone marrow depression), bone marrow response rate on day 33 (D33 BMR; M1: ratio of primitive cells to naive cells <5%, M2: 5%–25%, M3: >25%, M4: bone marrow depression), and measurable residual disease status on days 15 and 33 (D15 MRD and D33 MRD). Flow cytometry was employed to detect MRD. The standard criterion for a negative MRD result after the first induction therapy was MRD level <0.01%. The follow-up end date was 30 June 2022. The duration of EFS was recorded, and it was defined as the time from the date of diagnosis to the failure of induction therapy, disease recurrence, the occurrence of a secondary tumor, or patient death.

### 2.2 Data processing

#### 2.2.1 Statistical methods

The collected data were processed using R software (version 4.4.1). The development and evaluation of machine learning models were performed using the *mlr3verse, tidyverse*, and *mlr3extralearners* packages. Normally distributed quantitative data were represented as mean ± standard deviation, and a t-test was used for inter-group comparison. Non-normally distributed quantitative data were represented by the median (first quartile, third quartile), and the non-parametric Mann–Whitney U rank-sum test was used for inter-group comparison. Categorical data were presented as number (%), and the comparison between groups was conducted using the χ^2^ test. The Cox proportional hazards regression model was validated using the Logrank test. A two-sided P < 0.05 was considered statistically significant.

#### 2.2.2 Preprocessing of feature variables

In the univariate selection process, variables with P < 0.1 were selected. Subsequently, correlation analysis was conducted on these variables using the *recipes* package. If the correlation coefficient between two features exceeded 0.7, one of the features was removed to avoid multicollinearity affecting the model. To improve model efficiency and prevent overfitting, zero-variance features were removed.

#### 2.2.3 Machine learning model training and tuning

The data were divided into a training and a test set (8:2 ratio) using the stratified random sampling method to ensure that there were no significant differences in characteristics and results between the two sets (Supplementary Figure S2 P = 0.83). Models were built using the training set and validated on the test set. When building machine learning models, grid search or random search methods were used for hyperparameter tuning, and the optimal parameter set was determined by searching through predefined parameter combinations.

##### 2.2.3.1 Random forest

The number of generated decision trees was set between 200 and 500, with 2–10 features considered for each node and a minimum sample size of 2–21 for leaf nodes. Grid search was used for optimization, the resolution was set to 5, and the model was evaluated using five-fold cross-validation. Using the survival index C-index as the evaluation metric, without setting a stop condition, the tuner was allowed to continue running until all combinations had been tried. The resampling method for tree growth was “swor”, the splitting rule was random Logrank, and the number of random split points was set to 10.

##### 2.2.3.2 Gradient boosting machine

For the GBM model, the number of boosting iterations was set between 100 and 500. The maximum depth of each tree was 1–3, and the minimum number of observations for terminal nodes was 5–7. The learning shrinkage rate of each tree’s contribution to the final prediction result was set between 0.001 and 0.1. The Kaplan–Meier method was used for survival analysis estimation, with the Cox proportional hazards regression model as the formula type. We used the C-index as the evaluation metric and grid search as the adjustment method, with a resolution of 3; model performance evaluation adopted five-fold cross-validation.

##### 2.2.3.3 Extreme gradient boosting

For the XGBoost model, the number of iterations was set between 50 and 800, the maximum tree depth was 1–20, the learning rate was 10^−6^ to 1, and it used “Kaplan” as the estimator and “ph” as the model form. An automatic tuner was built for parameter search to effectively determine the optimal parameter combination, thereby improving model performance and prediction accuracy. Four parameter combinations were searched each time, and CV cross-validation was used to evaluate the model. The C-index was used as the evaluation index, and the evaluation stopping conditions were adjusted. A total of 40 evaluations were conducted.

### 2.3 Model validation

The effectiveness of the model was evaluated using cases from the First Affiliated Hospital of Guangxi Medical University as external data (n = 36). The model’s performance was evaluated through the C-index and the area under the receiver operating characteristics curve (AUROC).

## 3 Results

### 3.1 Baseline data and univariate screening

**FIGURE 1:**
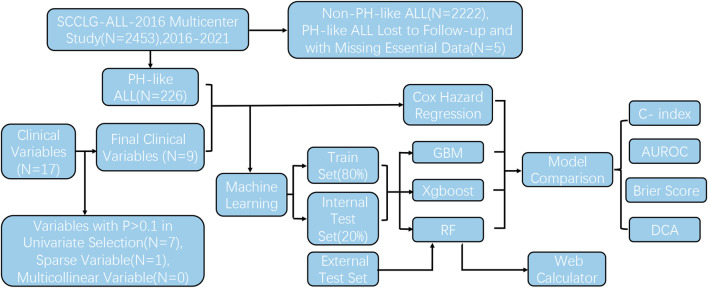
Consort diagram for Ph-like ALL selection, chosen clinical variables, and study workflow.

From October 2016 to May 2022, 2,453 children were treated and followed up according to the SCCLG-ALL-2016 guidelines for ALL (version 20191101.5). A total of 231 patients were diagnosed with Ph-like ALL. Five were excluded because of loss to follow-up and the absence of important clinical features. Ultimately, 226 Ph-like ALL patients were included (Figure 1). D15 BMR, D33 BMR, D15 MRD, D33 MRD, and the number of bone marrow blasts at diagnosis were significantly higher in the event-occurring group than in the non-event group (Table 1). Based on the statistical results, age, white blood cell count, platelet count, proportion of blasts, ET, PR, D15 MRD, D33 MRD, D15 BMR, and D33 BMR were identified as potentially correlated with EFS (P < 0.1). These feature variables were then used for subsequent Cox hazard regression or machine learning model development.

**TABLE 1:** Patient features.

Fetures	Model set			Validation set	
	Number of patients (%)/Mean (Media)	Patients with EFS(%)	P Value	Number of patients (%)/Mean (Media)	Patients with EFS(%)
Total Number	226	192 (84.956)		36	28 (77.78)
Gender			1		
Male	129 (57.1)	110 (57.3)		16	13 (46.2)
Female	97 (42.9)	82 (42.7)		20	15 (53.8)
Chromosomal morphology typing			0.114		
Normal	180 (79.6)	149 (77.6)		26 (72.2)	22 (78.6)
Abnormal	46 (20.4)	43 (22.4)		10 (27.8)	6 (21.4)
ET			0.065		
No	212 (93.8)	183 (95.3)		34 (94.4)	26 (92.9)
Yes	14 (6.2)	9 (4.7)		2 (5.6)	2 (7.1)
IK6 mutation			0.352		
No	113 (50.0)	99 (51.6)		19 (52.8)	16 (57.1)
Yes	113 (50.0)	93 (48.4)		17 (47.2)	12 (42.9)
ABL fusion			1		
No	183 (81.0)	155 (80.7)		28 (77.8)	23 (82.1)
Yes	43 (19.0)	37 (19.3)		8 (22.2)	5 (17.9)
Kinase pathway abnormality			0.897		
No	134 (59.3)	113 (58.9)		20 (55.6)	19 (67.9)
Yes	92 (40.7)	79 (41.1)		16 (44.4)	9 (32.1)
Immunophenotype			0.609		
Pro-B	9 (4.0)	8 (4.2)		2 (5.6)	1 (3.6)
Common B	194 (85.8)	166 (86.5)		27 (75)	25 (89.2)
Pre-B	9 (4.0)	6 (3.1)		3 (8.3)	1 (3.6)
Mixed phenotype	1 (0.4)	1 (0.5)		0	0
Immature-B	13 (5.8)	11 (5.7)		4 (11.1)	1 (3.6)
PR			0.081		
Sensitive	202 (89.4)	175 (91.1)		31 (86.1)	27 (96.4)
Insensitive	24 (10.6)	17 (8.9)		5 (13.9)	1 (3.6)
D15BMR			0.005		
M1	163 (72.1)	143 (74.5)		29 (80.6)	24 (85.7)
M2	35 (15.5)	31 (16.1)		1 (2.8)	1 (3.6)
M3	22 (9.7)	13 (6.8)		5 (13.8)	3 (10.7)
M4	6 (2.7)	5 (2.6)		1 (2.8)	0
D33BMR			<0.001		
M1	211 (93.4)	184 (95.8)		33 (91.6)	27 (96.4)
M2	4 (1.8)	2 (1.0)		2 (5.6)	1 (3.6)
M3	2 (0.9)	0 (0.0)		1 (2.8)	0
M4	9 (4.0)	6 (3.1)		0	0
Age year	4.20 [1.65, 7.30]	4.00 [2.60, 6.90]	0.006	3.25 [2.85,8.25]	3.3 [2.5,5.4]
WBC 10^9^/L	12.02 [4.84, 42.38]	10.20 [4.56, 36.47]	0.009	8.615 [4.18,22.233]	7.81 [3.46,12.55]
Blasts %	38.50 [6.00, 75.00]	32 [4.00, 67.50]	<0.001	22.3 [4.825,45.5]	17 [4.3,30.4]
HB g/L	72.38 ± 20.93	72.04 ± 21.50	0.559	70.5 ± 22.75	59.1 ± 23.608
PLT 10^9^/L	45.00 [17.12,100.25]	46.5 [18.15, 113.25]	0.095	46.5 [13.57,120.92]	57 [21,153]
D15MRD %	0.92 [0.08, 8.60]	0.68 [0.07, 4.73]	<0.001	0.505 [0.065,5.225]	0.31 [0.05,1.12]
D33MRD %	0.00 [0.00, 0.02]	0.00 [0.00, 0.01]	0.001	0 [0,0]	0 [0,0]

### 3.2 Feature variable preprocessing

No multicollinearity was detected among the included features (Supplementary Figure S1). However, D33 BMR was removed as a sparse variable with zero-variance.

### 3.3 Cox hazard regression model

**FIGURE 2:**
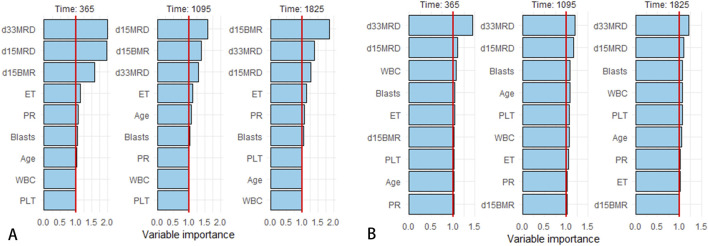
Variable importance rankings for 1/3/5-year predictions. **(A)** Variable importance in Cox proportional hazards regression model. **(B)** Variable importance in RF model.

The final prediction model indicated that D15 BMR, D15 MRD, D33 MRD, and ET were statistically significant factors (Logrank score = 65.81, P < 0.01—Table 2), and the C-index was 0.515. In the test set, the predictive accuracies of EFS at 1, 3, and 5 years after the diagnosis of Ph-like ALL were 0.661, 0.538, and 0.529, respectively. The importance of each feature is shown in Figure 2A.

**TABLE 2:** Cox proportional hazards regression analysis.

Feature	Regression coefficient β	5-year EFS HR (95% CI)	SE	Z value	p value
ET					
No		1.00			
Yes	1.827	6.218 (1.74–22.247)	0.65	2.81	0.005
PR					
Sensitive		1.000			
Insensitive	1.177	3.245 (0.935–11.267)	0.635	1.854	0.064
D15BMR					
M1		1.000			
M2	−2.561	0.077 (0.014–0.441)	0.889	−2.88	0.004
M3	−3.153	0.043 (0.005–0.357)	1.083	−2.912	0.004
M4	−9.160	0.002 (0.001–0.007)	2.138	−4.284	0
Age	0.004	1.042 (0.929–1.168)	0.058	0.700	0.484
D15MRD	0.079	1.082 (1.05–1.121)	0.018	4.425	0
D33MRD	0.067	1.069 (1.04–1.099)	0.014	4.74	0
blasts	0.008	1.008 (0.992–1.024)	0.008	0.984	0.325
PLT	−0.003	0.999 (0.994–1.004)	0.003	−0.458	0.647
WBC	0.002	1 (0.995–1.005)	0.003	0.081	0.936

### 3.4 Machine model parameter tuning

**FIGURE 3:**
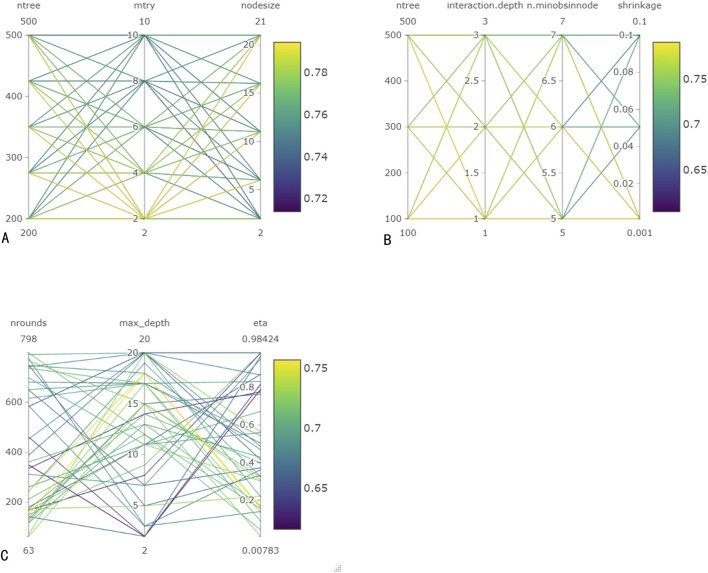
Hyperparameter tuning for machine learning models. **(A)** Hyperparameter tuning for RF. **(B)** Hyperparameter tuning for GBM. **(C)** Hyperparameter tuning for Xgboost.

As illustrated in Figure 3, the optimal prediction model of the RF algorithm has a C-index of 0.794. The model consisted of 350 decision trees, with each node considering two features and each leaf node containing at least 21 samples. The out-of-bag continuous ranking probability score of this model was 0.097, with a performance error of 0.246. In the GBM model, when there were 100 decision trees, the tree depth was 1, the leaf nodes contained at least five samples, the shrinkage value was 0.001, and the C-index reached its highest value of 0.79. In the XGBoost model, after 150 iterations, the maximum C-index was 0.757, the maximum tree depth was 18, and the tree weight update amplitude control parameter η for each iteration was 0.158.

### 3.5 Model comparison

**FIGURE 4:**
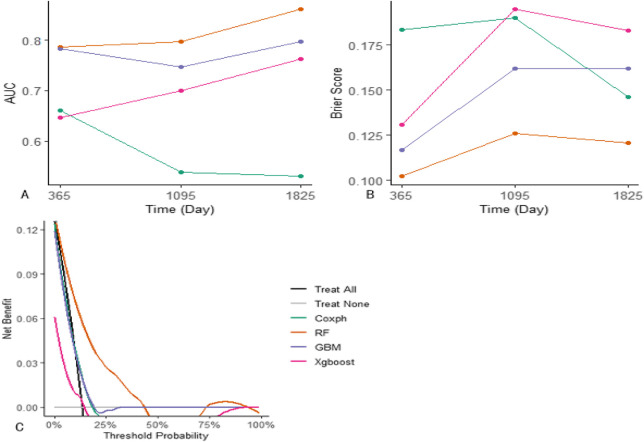
Comparison between machine learning models and Cox proportional hazards regression model. **(A)** Evaluation of models’ AUC on the training set: RF model demonstrates superior performance in AUC for 1/3/5-year predictions, achieving values of 0.787, 0.797, and 0.861, respectively. GBM model attains AUC values of 0.784, 0.747, and 0.789 for the corresponding time points, while Xgboost achieves 0.646, 0.7, and 0.764. In contrast, Cox proportional hazards regression model achieves AUC values of 0.661, 0.538, and 0.529 for the respective prediction intervals. **(B)** Assessment of models’ Brier scores: RF model yields the lowest brier scores for 1/3/5-year predictions, recording values of 0.102, 0.126, and 0.121, respectively. GBM model follows with Brier scores of 0.117, 0.162, and 0.162, and Xgboost with scores of 0.131, 0.195, and 0.183. Cox proportional hazards regression model exhibits Brier scores of 0.183, 0.19, and 0.146 for the corresponding prediction intervals. **(C)** Comparative analysis of models’ DCA curves: RF model demonstrates the largest area under the DCA curve across predictions, indicating its superior performance in DCA.

**FIGURE 5:**
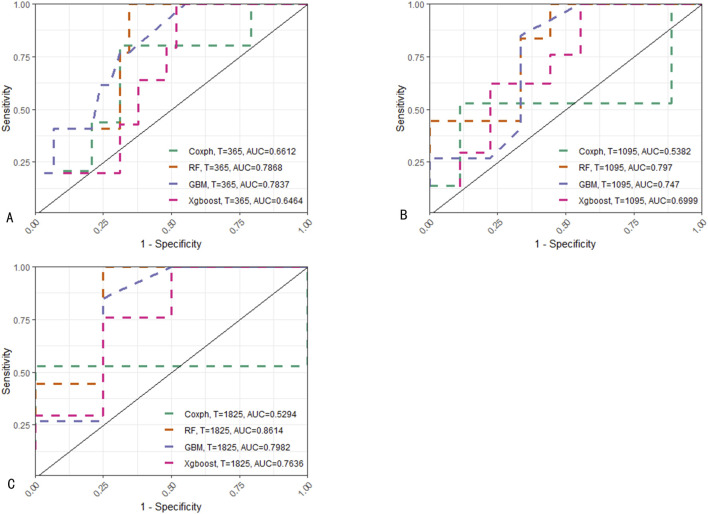
ROC comparison of predictions on the training set at different time points among various models. **(A)** Predictions of four models for 1-year EFS. **(B)** Predictions for 3-year EFS. **(C)** Predictions for 5-year EFS. For predictions at the aforementioned three time points, RF consistently demonstrates the best performance among the models.

The four models were evaluated using receiver operating characteristic (ROC) curves, AUROC, Brier scores, and decision curve analysis (DCA) (Figures 4, 5). The RF model outperformed other models in these evaluation metrics, particularly in predicting EFS. In the test set, the RF model achieved a prediction accuracy of approximately 80% for EFS at 1, 3, and 5 years after the diagnosis of Ph-like ALL, which was significantly higher than those of the traditional Cox regression, GBM, and XGBoost algorithms. The RF model had the lowest Brier score, indicating relatively high prediction accuracy. In DCA curve analysis, the RF model had the largest area under the curve, suggesting that using the RF model to predict Ph-like ALL can yield maximum benefits.

### 3.6 Analysis of feature variables in the random forest model

**FIGURE 6:**
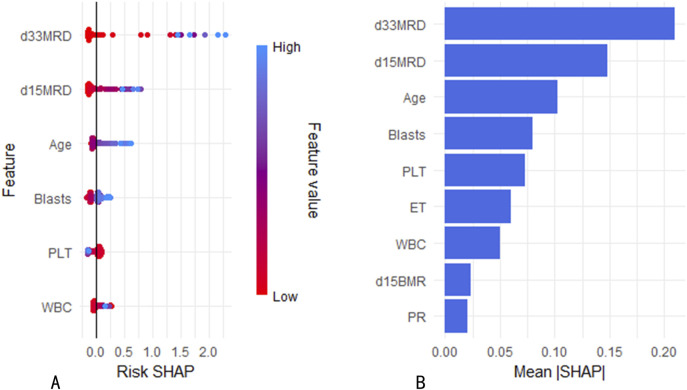
**(A)** Risk SHAP values for continuous variables in the RF model. **(B)** Mean SHAP values of variables in the RF model.

**FIGURE 7:**
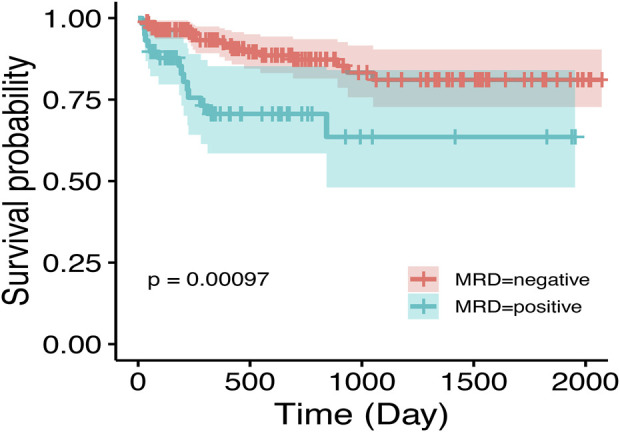
Impact of D33 MRD on event-free survival in Ph-like ALL. MRD negativity is achieved post-first induction, defined as MRD <0.01%.

SHapley Additive exPlanations (SHAP) is a method used to interpret the output results of machine learning models. This method assigns a contribution score to each feature by assessing its impact on the model output. In the RF machine learning model, variables such as D33 MRD, D15 MRD, and the proportion of blasts were correlated with the prediction of EFS at 1, 3, and 5 years after the diagnosis of Ph-like ALL (Figure 2B). According to the SHAP risk analysis, patients with MRD and a low proportion of blasts after treatment showed higher EFS rates. High levels of MRD and a high proportion of blasts increased the probability of adverse events after diagnosis (Figure 6A). Survival analysis based on whether MRD turns negative also confirmed that D33 MRD was an important clinical indicator affecting prognosis (Logrank test, χ^2^ = 8.894, P < 0.01—Figure 7). Notably, age significantly influenced the prediction of 3-year EFS (Figure 2B), with older children having a higher probability of adverse events than younger patients. Additionally, lower levels of platelet count were found to trigger adverse events (Figure 6A). Figure 6B shows the average SHAP values of individual clinical features in the RF model for prediction accuracy. As it shows, compared with the Cox proportional hazards regression model, the impacts of ET and D15 BMR on the model’s predictive capabilities were relatively small, as indicated by their SHAP values.

### 3.7 Model validation

**FIGURE 8:**
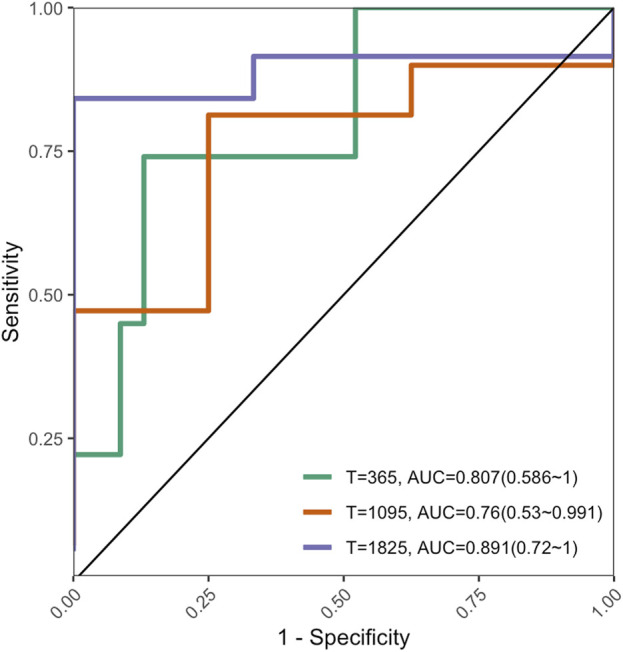
Performance of AUROC in external validation dataset.

The C-index of the external validation dataset was 0.753 (95% CI: 0.55–0.904, P < 0.01); Figure 8 shows the AUROC performance at 1, 3, and 5 years.

## 4 Discussion

In the Ph-like ALL data of our collaborative group, the clinical features evaluated by the model include common molecular genetic abnormalities, age, MRD, BMR, and immunophenotyping in Ph-like ALL. Ultimately, the clinical features that play a major role in accurate classification and prediction are MRD, age, and blasts rather than molecular genetic abnormalities. Among these features, MRD has the most significant impact on the model. There have been many studies and evaluations regarding the impact of MRD response-based intensified treatments on the survival prognosis of Ph-like ALL. A research team from the American Anderson Cancer Center reported that MRD negativity after induction therapy has no significant impact on the long-term survival of adult patients with Ph-like ALL (31). A study by St. Jude’s Children’s Research Hospital found that risk-oriented treatment based on MRD could significantly improve the poor prognosis of Ph-like ALL (Jeha et al., 2021). Cho et al. (2021) also advocated MRD monitoring as a criterion for further treatment assessment, especially in determining whether patients with Ph-like ALL require allogeneic hematopoietic stem cell transplantation, highlighting its significant evaluative value. In the RF survival prediction model of this study, both D33 MRD and D15 MRD made significant contributions to 1-, 3-, and 5-year survival predictions, with D33 MRD being particularly prominent.

Published studies have shown that specific genetic molecular changes in Ph-like ALL may have varying effects on treatment outcomes. Studies on adult patients have shown that compared to patients without related rearrangements, CRLF2 or JAK2/EPOR rearrangements are associated with lower survival rates (Roberts et al., 2017b). JAK mutations, as common genetic molecular abnormalities in Ph-like ALL, are believed to be associated with poor prognosis (Mullighan et al., 2009; Herold et al., 2017). Another study on adult Ph-like ALL patients found that CRLF2 overexpression is associated with poor prognosis, with a 5-year survival rate of less than 20% (Roberts et al., 2014a; Jain et al., 2017). However, in research on a cohort of children with Ph-like ALL, van der Veer et al. (2013) reported that IKZF1 deletion rather than CRLF2 overexpression was one of the factors leading to poor prognosis. In Ph-like ALL cases, some studies have shown that IK6 deletion has an independent prognostic impact, tripling the risk of treatment failure (Hu et al., 2023; Stanulla et al., 2018), while others suggest that IKZF1 deletion is not significantly correlated with disease relapse or long-term survival (Cho et al., 2021). These different or contradictory outcomes may be related to variations in patient age, race, sample size, and treatment protocols. In the RF prediction model based on the Ph-like ALL data of our collaborative group, IK6 deletion and molecular genetic changes involving kinase pathways, such as CRLF2 rearrangement or overexpression, JAK mutations, or JAK fusion proteins, have no significant impact on the prognosis of Ph-like ALL. This may be related to the targeted therapy or intensified chemotherapy regimen received by Ph-like ALL patients in our group’s treatment plan, which eliminated the adverse effects of these genetic molecular abnormalities on EFS, thus demonstrating the effectiveness of these treatment methods from another perspective.

In the RF model’s prediction of 3-year EFS, age is also one of the factors affecting EFS. In Ph-like ALL cases with adverse events, the average age at diagnosis was higher than in cases without events. As shown in Figure 4, as age increases, the adverse effects on survival gradually increase, and the probability of a decrease in survival rate also increases. In view of the increasing incidence rate of Ph-like ALL before adulthood and the diverse changes in genetic molecules, new fusion genes or gene mutations are constantly being found. When cases cannot be clearly classified as Ph^+^ ALL, mixed lineage leukemia rearrangement, ETV6-RUNX1 fusion, and other subtypes, the diagnosis of Ph-like ALL should be emphasized. Moreover, during the treatment process for older children with Ph-like ALL, MRD should be closely monitored, and the treatment plan and intensity should be actively adjusted to effectively reduce MRD levels and improve EFS rates.

The widespread application of machine learning techniques such as RF, GBM, and XGBoost in medical research has demonstrated excellent performance in survival prediction, particularly when processing high-dimensional data, indicating significant potential. Currently, machine learning is widely employed in the diagnosis and prognosis of numerous diseases (Asadi et al., 2021; Walker et al., 2022; Gel et al., 2023). In addition to the above algorithms, this study also constructed machine learning models using K-nearest neighbors, Lasso regression, and support vector machine methods. However, the performance of these three algorithms was suboptimal. RF, an ensemble algorithm, is composed of multiple decision trees. The results of each tree are derived from randomly sampled training instance sets and feature subsets. Compared with single decision trees, this approach is more robust and has a greater ability to prevent overfitting. During disease diagnosis or prognosis evaluation, RF can identify factors such as genes, biomarkers, and clinical features that exhibit significant differences (Barberis et al., 2022; Ghosh and Cabrera, 2022). For each feature, RF assesses its importance by calculating the average impurity reduction of that feature across all decision trees. The RF prediction model constructed in this study can predict the EFS probability of Ph-like ALL relatively accurately. After evaluation from multiple dimensions, such as C-index, ROC curve, AUROC, Brier score, and DCA curve, it was found that the RF prediction performance for Ph-like ALL is superior to that of other machine learning models and traditional Cox proportional hazards regression models. In data analysis, the Cox proportional hazards regression model identifies D15 BMR, ET, and MRD as independent risk factors influencing EFS. In the RF model, the contributions of D15 BMR and ET gradually decline as survival time lengthens. Based on the unique distribution patterns observed in the data of this study, machine learning models demonstrate more accurate predictive capabilities. It is therefore evident that the RF model is proficient in constructing intricate models to analyze multifactorial impacts on treatment outcomes. Consequently, the constructed RF model was deployed as a web-based calculator (https://songxiaodan03.shinyapps.io/RFpredictionmodelforPHlikeALL/). It can offer crucial references for tailoring personalized treatment strategies for patients.

However, the analysis in this study has certain limitations. In the retrospective analysis, patients with incomplete medical records and a small number of Ph-like ALL patients were excluded from the study because of treatment abandonment, poor treatment response, and failed referral or follow-up. This may introduce bias in the sample selection process. Additionally, since Ph-like ALL is not caused by a single molecular genetic mechanism, a small number of cases may not involve kinase pathway abnormalities or may result from new gene fusion. Such cases typically require expensive tests, such as panoramic gene sequencing, for diagnosis. However, there are variations in the completion rates of expensive tests, such as whole-exome sequencing and panoramic gene sequencing, among the 13 hospitals in the collaborative group located in regions with different economic levels. This inconsistency may result in delayed diagnosis and analysis of extremely rare Ph-like ALL subtypes.

## 5 Conclusion

In a big data context, the importance and feasibility of integrating machine learning models into precision medicine are apparent. When compared with linear models, machine learning models are capable of offering more precise and dependable predictions and judgments. This study found that integrated machine learning models outperform traditional models in prediction accuracy, providing new perspectives and tools for future research. This study underscores the substantial advantage of the RF model in prediction accuracy, highlights the evaluative value of MRD in predicting the prognosis of Ph-like ALL patients, identifies the key factors influencing the survival prediction of Ph-like ALL, and fully validates the capability of machine learning in disease survival prediction. The outcome of this research offers a significant reference for future precision medicine research based on big data and complex datasets. Based on these findings, an RF machine learning model can offer personalized assessments and treatment recommendations for Ph-like ALL patients. As technology advances, machine learning models are being used more extensively in clinical practice for diagnosis classification, prognosis evaluation, and other tasks based on various clinical features.

## Data Availability

The datasets generated and/or analyzed during the current study are not publicly available because they involve human patient privacy and ethical restrictions. The data analyzed in this study were obtained from the SCCLG. Restrictions apply to the availability of these data, which were used under license for this study. Data are available from the authors upon reasonable request with the permission of SCCLG.

## Supplementary material

SUPPLEMENTARY FIGURE S1 Correlation analysis of clinical variables.

SUPPLEMENTARY FIGURE S2 Survival analysis comparison of training set and test set data.

## Abbreviations

SCCLG, South China Children’s Leukemia Group; AUROC, area-under-the-receiver operating characteristics curve; ALL, acute lymphoblastic leukemia; Ph-like ALL, Philadelphia chromosome-like acute lymphoblastic leukemia; MRD, minimal residual disease; RF, random forest; GBM, gradient boosting machine; XGBoost, extreme gradient boosting; ET, extramedullary tumor; PR, prednisone response; BMR, response rate of bone marrow; EFS, event-free survival; DCA, decision curve analysis; SHAP, Shapley additive explanations.
