# Supplementary figures and images for: Survival prediction for Philadelphia chromosome-like acute lymphoblastic leukemia by machine learning analysis: a multicenter cohort study
Source: Front Cell Dev Biol. 2025 Sep 18;13:1650810. doi: 10.3389/fcell.2025.1650810 (PMC12488647; doi:10.3389/fcell.2025.1650810)

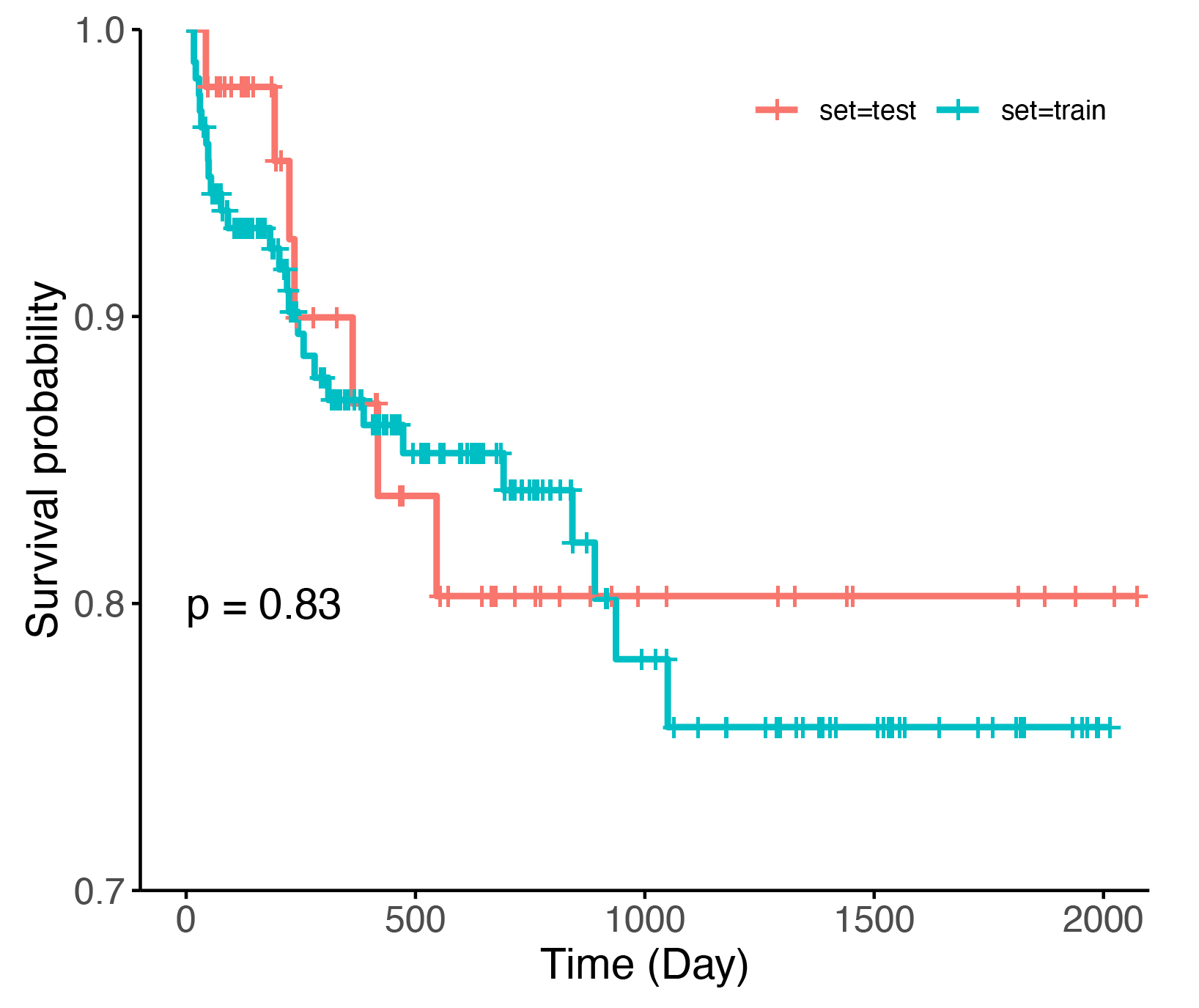

Supplement: Supplementary file 1 [file Image2.tif]

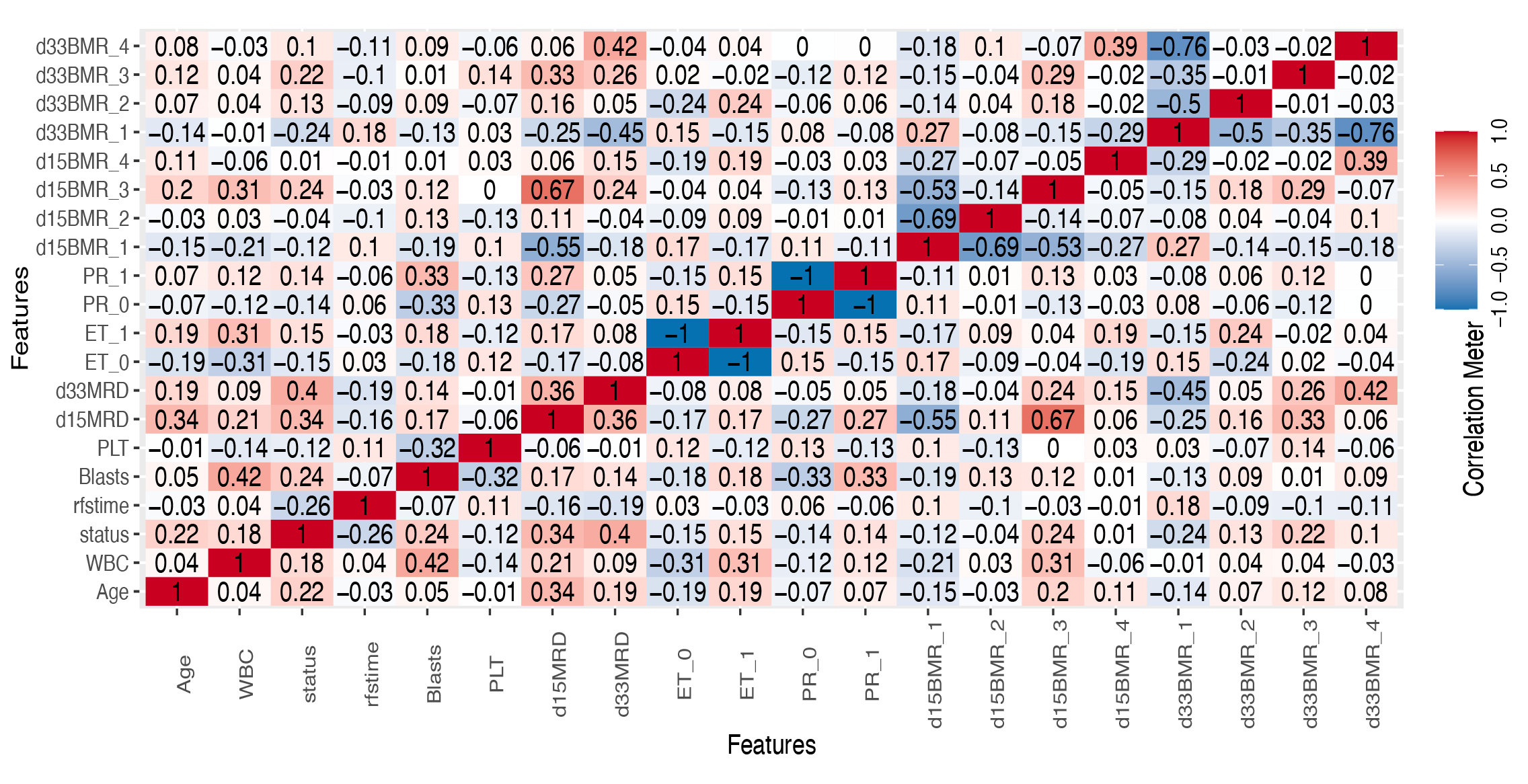

Supplement: Supplementary file 2 [file Image1.tif]
